# Clinical Outcomes of Extended-Spectrum Beta-Lactamase-Producing *Enterobacteriaceae* Infections with Susceptibilities among Levofloxacin, Cefepime, and Carbapenems

**DOI:** 10.1155/2018/3747521

**Published:** 2018-02-08

**Authors:** Kristy J. Walker, Young R. Lee, Amanda R. Klar

**Affiliations:** ^1^Hendrick Medical Center, 1900 Pine St., Abilene, TX 79601, USA; ^2^Department of Pharmacy Practice, Texas Tech University Health Sciences Center, School of Pharmacy, 1718 Pine St., Abilene, TX 79601, USA

## Abstract

**Purpose:**

Highly resistant Gram-negative bacterial infections are associated with high mortality. Increasing resistance to standard therapy illustrates the need for alternatives when treating resistant organisms, especially extended-spectrum beta-lactamase- (ESBL-) producing *Enterobacteriaceae*.

**Methods:**

A retrospective chart review at a community hospital was performed. Patients who developed ESBL-producing infections were included. Patients less than eighteen years old, who were pregnant, or who were incarcerated were excluded. The primary outcome was hospital mortality. The secondary outcomes included intensive care unit (ICU) mortality, ICU length of stay, and hospital length of stay.

**Results:**

113 patients with ESBL-producing infections met the criteria for review. Hospital mortality: carbapenem (16.6%), cefepime (0%), and levofloxacin (15.3%) (*p*=0.253). ICU mortality: carbapenem (4.5%), cefepime, (0%), and levofloxacin (3.7%) (*p*=0.616). Mean ICU and hospital length of stay: carbapenem (9.8 ± 16, 12.1 ± 1 days), cefepime (7.8 ± 6, 11.1 ± 10.5 days), and levofloxacin (5.4 ± 4.1, 11.1 ± 10.4 days) (*p*=0.805, 0.685). No predictors were clearly found between the source of infection and mortality.

**Conclusion:**

Cefepime or levofloxacin can be a potential alternative agent for infections with ESBL-producing *Enterobacteriaceae*, and larger clinical trials investigating these outcomes are warranted.

## 1. Introduction

Extended-spectrum beta-lactamase- (ESBL-) producing *Enterobacteriaceae* infections are increasingly identified with subsequent hospitalization [1, 2]. ESBL is a resistance mechanism in which the beta-lactam ring of antibiotics such as penicillins, cephalosporins, and aztreonam is hydrolyzed, inactivating the antibiotic. Increasing resistance was demonstrated in a 2013 Centers for Disease Control and Prevention (CDC) report, which included 26,000 ESBL-producing *Enterobacteriaceae* infections and 1700 deaths in the United States [3]. Traditionally, the treatment of choice for ESBL-producing *Enterobacteriaceae* infections has been the carbapenem class. However, trends in resistance demonstrate the need of conservative use of carbapenems and illustrate the need for alternative therapies against ESBL-producing organisms. Additionally, chromosomally mediated inducible resistance has manifested as porin loss, which decreases drug entry in strains already retaining high levels of AmpC [4]. Emerging incidence of community-acquired ESBL-identified infections has demonstrated a positive association with the increasing trend of carbapenem resistance in *Enterobacteriaceae*. Though prospective research demonstrating effectiveness of other therapies has been limited, there is emerging retrospective evidence in support of the use of cefepime, fluoroquinolones, and piperacillin/tazobactam in certain clinical situations [5–10].

It is of interest to the patient for organisms (*E. coli* being the most common to exhibit ESBLs) to be detected and reported to healthcare providers in a timely fashion for both appropriate treatment to be initiated and resulting morbidity and mortality to be significantly reduced. However, reporting of ESBL can be delayed by a few days due to the screening and identification process. Many microbiology laboratories are challenged by increasingly changing and complicated recommendations [11]. Minimum inhibitory concentrations (MICs) of *β*-lactamase-producing *Enterobacteriaceae* might be reported as high but are still in the susceptible range (i.e., “hidden resistance”) [7, 8]. If diagnostic microbiology laboratories are unable to adequately test for ESBL production, it could be argued that suspect cases of hidden resistance will go unrecognized by the microbiologists and clinicians, increasing the likelihood for adverse consequences [8–10]. Other data showed that carbapenem use when MIC is ≥4 mg/liter had outcomes of increased mortality compared to patients whose microbiological isolate MICs were lower, demonstrating carbapenem therapy limitations [12].

Because *Enterobacteriaceae* organisms are becoming increasingly resistant to standard therapy, treatment selected by interpretation of MICs of ESBL producers and by clinical outcome may offer therapeutic alternatives in more challenging situations [7, 12–14]. Appealing to the cause of antimicrobial stewardship, the fourth-generation cephalosporin cefepime could be a considerable alternative against ESBL-producing organisms and could shift trends for carbapenem use. Cefepime has shown to have greater, stable activity against ESBL (i.e., AmpC enzymes) compared to the other extended-spectrum cephalosporins [9, 10, 14]. Cefepime does not rely on porins for entry into the bacterial cell, making it useful in the down-regulated porin resistance mechanism [15, 16]. Appealingly, cefepime could be used in therapy for ESBL-EC and ESBL-K strains highly resistant to third-generation cephalosporins (including ceftazidime) and to aztreonam [14, 17–21]. Fluoroquinolones may also offer a place as alternative therapy in treating ESBL-producing *Enterobacteriaceae*. Though many bacteria are becoming increasingly resistant to fluoroquinolones, treatments with this class when susceptibilities allow have demonstrated improved inpatient mortality when compared to carbapenems [22–25]. A small retrospective review with in vitro samples indicated no difference in inpatient mortality with levofloxacin when comparing low, intermediate, and high MICs. However, they did demonstrate trends for a shorter improved mortality and shorter length of stay in the lower MIC group [22].

The objective of this study is to evaluate clinical outcomes among levofloxacin, cefepime, and carbapenem used for ESBL-producing *Enterobacteriaceae* infections. Additional objectives include identifying risk factors that can help tailor antibiotic therapy.

## 2. Methods

This is a retrospective chart review conducted at one 522 (including 22 ICU) bed tertiary community medical center. Patient charts were included from January 1, 2012, to September 30, 2015. Records and profiles of discharged patients who received cefepime, levofloxacin, or carbapenem for ESBL pathogens within the specified time span were identified by the microbiology lab department. This study received approval by the Texas Tech University Health Sciences Center Institutional Review Board. Individuals with ESBL-producing *Enterobacteriaceae* infections were identified, who also waived the need for informed consent. Eligible patients fulfilled each of the following criteria: (1) aged 18 years or older, (2) clinically diagnosed with the ESBL-producing *Enterobacteriaceae* isolate demonstrated on culture, (3) received empirical treatment with cefepime, levofloxacin, or carbapenem for at least 48 hours after initial cultures had been drawn, and (4) admitted for inpatient treatment. Ninety days prior to culture, risk factors were identified. The researchers took no part in the conduct of the study, nor did they play a role in the analysis of the data.

An infection occurring in the ICU was defined as the patient being present in the ICU when the culture was identified for the Gram-negative organism. Infectious and other diagnoses were identified by ICD-9 codes in the patient chart. Acute renal failure is identified when the serum creatinine is increased by 50% or more from the patient's baseline serum creatinine on the onset of infection. Included in this were patients with a past medical history of chronic kidney disease (CKD) noted in the chart. Antimicrobial therapy administered after diagnosis was regarded as empirical treatment, and therapy administered afterward was defined as definitive therapy.

This single medical center has a centralized clinical microbiology laboratory which processes 96,000 samples annually. At this laboratory, bacteria can be identified down to a genus species, and the susceptibilities are further outlined by predefined antimicrobials. An automated broth microdilution system (MicroScan; Siemens AG, Germany) allows for susceptibility reporting, and an analysis is conducted in accordance with the Clinical and Laboratory Standards Institute (CLSI) criteria [26]. To identify ESBL-producing bacteria, isolates are processed using the MicroScan WalkAway system. This system initially screens for ceftazidime and cefpodoxime resistance, which is subsequently confirmed on demonstration of synergy between these two antibiotics and additionally by clavulanic acid on disc synergy testing. The interpretation followed the current breakpoints recommended by the CLSI. The severity of underlying medical illness was calculated by the Elixhauser scoring system [27].

The primary study outcome was hospital mortality. The secondary outcomes were ICU mortality, ICU length of stay, and hospital length of stay.

The following baseline characteristics were also collected: age, gender, type of infection, source of infection, culture species, and sensitivities.

All analyses were performed using Strata Version 13.1 (StataCorp, College Station, TX). Descriptive statistics were used to quantify patient characteristics, as well as for analysis of the primary and secondary outcomes. Categorical variables were expressed as percentages of total numbers of patients analyzed. Continuous variables were expressed as mean values ± SDs. The Kruskal–Wallis test was used to compare differences between groups. Multivariate regression analyses were used to identify risk factors that may have association with clinical outcomes. Data are summarized as *n* (%). Adjusted odds ratios (ORs) are presented with 95% confidence intervals and were calculated using multivariate logistic regression. OR *p* values correspond to adjusted ORs.

## 3. Results

### 3.1. Baseline Characteristics

In this review, 113 patients with ESBL-producing *Enterobacteriaceae* infections were successfully identified at the community hospital during the 3-year study period (Table 1). 66 patients were in the carbapenem group, 21 in the cefepime group, and 26 in the levofloxacin group. Table 1 shows the characteristics of the patients at baseline. The mean age of the patients was 69.8 years, and 60% were female. There were 77.3% patients in the carbapenem group, 85.7% in the cefepime group, and 88.9% in the levofloxacin where the pathogen was identified as *E. coli*. The predominant infection treated was urinary tract infection (UTI) with 66.7% in the carbapenem group, 57.1% in the cefepime group, and 70.4% in the levofloxacin group. Additionally, 7.6% in the carbapenem group, 0% in the cefepime group, and 7.4% in the levofloxacin group were treated for intra-abdominal infection. Therefore, no patients were treated with cefepime for intra-abdominal infection.

Furthermore, 15.2% in the carbapenem group, 28.6% in the cefepime group, and 7.4% in the levofloxacin group were treated for skin and soft tissue infection (SSTI). Of note, carbapenem and levofloxacin groups were treated for over 60% hospital-acquired or healthcare-associated infections, while the cefepime group was treated for 61.9% community-acquired infections.

### 3.2. Primary and Secondary Outcomes

Hospital mortality and ICU mortality were not different in all three groups. No one died in the cefepime group even though there was no statistical difference (Table 2). ICU length of stay was shorter in the cefepime and levofloxacin groups, by 2.0 and 4.4 days, respectively. Hospital length of stay was shorter by 1 day in the cefepime and levofloxacin groups compared to the carbapenem group, although this was not statistically different.

### 3.3. Multivariate Analysis

There were no predictors found between the source of infection and mortality (Table 3).

## 4. Discussion

There is controversy in recent literature regarding alternative therapies against infections due to ESBL-producing *Enterobacteriaceae*. This study has outlined outcomes for a rural healthcare system where cefepime and levofloxacin were used as alternatives for treatment of infections.

Studies demonstrate improved inpatient mortality when susceptibilities have allowed treatment with fluoroquinolones such as ciprofloxacin and levofloxacin [22–24]. However, most of these studies only studied bacteremia as the primary type of infection, whereas our study allowed for all types of infection. Furthermore, these studies were following the earlier break points of MIC susceptibility established by the CLSI. Antimicrobial sensitivity to cefepime and levofloxacin among *Enterobacteriaceae* is defined by the 2014 CLSI update as an MIC of less than or equal to 2 *μ*g/mL [26]. A randomized control trial by Seo et al. demonstrated a high treatment failure comparing cefepime, piperacillin/tazobactam, and ertapenem in treatment of ESBL-producing *E. coli* infection [28]. However, the baseline characteristics of patients in the study were not well aligned. The sex differences between cefepime and the other 2 groups varied, as well as the average age of patients in the cefepime group was approximately 10 years greater than the piperacillin/tazobactam and ertapenem groups. Our study, however, showed similar reporting between ages and sexes.

Previous literature showed an association between clinical outcomes and MICs. These studies have suggested that a low MIC value is positively associated with better clinical outcomes versus a higher MIC [9, 10, 17, 19–22]. Recent literature has trended favorably from a sensitivity-based approach to an MIC-based approach in settings where ESBL producers are endemic [29–31]. Studies have shown that cefepime and levofloxacin can both favorably achieve pharmacodynamic targets against isolates of fully susceptible *Enterobacteriaceae* at lowered MIC susceptibilities [11, 12, 15, 18, 22]. There have been underlying issues with studies regarding the lack of clinical experience using these medications, as well as the conflicting reports of clinical outcomes in the literature when studying treatments for ESBL-producing *E. coli* infection. Additional research would help demonstrate that drug target attainment in ESBL-producing *Enterobacteriaceae* with cefepime and levofloxacin will achieve adequate drug target levels when using the recent updates for susceptible breakpoints for cefepime and levofloxacin.

Additionally, the doses of cefepime used in the study may have been subtherapeutic, whereas our doses were reviewed for therapeutic efficacy and we also took into account if renal dose adjustments had been made [28]. Lower doses of cefepime have been associated with treatment failure and higher mortality [28, 32]. Literature suggests that higher maximal doses of cefepime, even when renally adjusted, may be more beneficial than standard cefepime dosing [32]. Because time above MIC is critical for clinical success of cefepime dosing, our study evaluated if the dose was appropriate based on the infection type and renal function [32, 33].

Previous retrospective chart review had observed a trend of lower 30-day mortality in the fluoroquinolone group compared to the carbapenem group, although not statistically significant (OR 4.53; 95% CI 0.98–21) [23]. Similarly, a higher 30-day mortality in the cefepime group was demonstrated when CLSI breakpoints were 8 or greater (OR 7.1; 95% CI 2.5–20.3) [21]. However, unlike our study, these two trials only looked at bacteremia infection and had used previously defined CLSI breakpoints for cefepime. Although this study did not find a statistically significant difference either, when using a lower MIC breakpoint, there was a trend for lower mortality for both alternative therapy groups. Thereby, it was suggested that cefepime therapy was limited for bacteremia caused by ESBL-producing *Enterobacteriaceae* organisms when cefepime MIC was less than or equal to 1 *μ*g/mL [21, 23]. Additional comparisons of length of hospital stay had shown that higher MIC resulted in increased length of stay of 5.67 days (95% CI 0.77–10.62 days; *p*=0.02) with levofloxacin [22]. Similar to other studies, our study was limited by allowing other antimicrobial agents that could have altered the outcomes, although matched group analysis was applied to our study to offset this limitation.

Our study did not identify a statistically significant difference in hospital mortality between patients who were receiving treatment with carbapenem and those who received an alternative treatment. However, this study did find a trend between carbapenem therapy and an increased risk of mortality and alternative therapy and a decreased risk of mortality. It follows that there might be greater in vitro activity in favor for cefepime, specifically for not being induced by the AmpC gene. Interestingly, no one died in the cefepime group even though there was no statistical difference. This could be explained by many community-acquired infections in the cefepime group, but this suggests that cefepime can be a good treatment option in ESBL community-acquired infections. Carbapenem therapy was associated with a trend for longer hospital stay than with cefepime therapy. This association is not entirely clear but is possibly related to increased level of severity of illness among patients receiving carbapenem therapy. More patients with septic shock and pneumonia were included in the carbapenem group, and more community-acquired infections were in the cefepime group. Although unable to demonstrate an association between mortality and source of infection for patients receiving cefepime or levofloxacin, it is possible that this study was underpowered to identify an association. Although unable to demonstrate an association between increased MIC of cefepime and mortality among patients receiving cefepime monotherapy, it is possible that the results might have been diluted due to the inability to detect definitive MIC values.

Our study had a few strengths. First, this research was specifically looking at clinical outcomes for the hospital and ICU. Additionally, this study allowed for identification of multiple *Enterobacteriaceae* organisms. This study also allowed for several types of infection. Up until this point, previous literature had been limited to specific types of *Enterobacteriaceae* (e.g., only *E. coli*) and had only studied outcomes in cases of bacteremia or UTI.

This study did have several limitations. First, there was a low sample size for the cefepime and levofloxacin groups compared to the carbapenem group. This is likely due to the physician's preference for the carbapenem group. Carbapenem therapy is used in this healthcare setting predominantly for ESBL-producing *Enterobacteriaceae*. Additionally, it was challenging to determine the exact MIC, as the MIC reported used automated log_2_ dilution. In previous literature, an MIC of 1 or less proved to have a positive association between cefepime therapy and mortality. If this study had been able to detect an exact MIC of 1, there might have been a better detection for outcomes. Finally, there potentially was a physician selection bias for cefepime therapy in patients receiving treatment for community-acquired ESBL-producing infections.

## 5. Conclusion

Results from this study suggest that either cefepime or levofloxacin can be a potential alternative agent for infections with ESBL-producing *Enterobacteriaceae* for hospitalized patients when isolates are susceptible. Further studies with larger sample size and well-balanced type of infections between groups are warranted.

## Figures and Tables

**Table 1:** Baseline characteristics.

Characteristics	Carbapenem (*n* = 66)	Cefepime (*n* = 21)	Levofloxacin (*n* = 26)	*p* value
*Demographics*				
Age (years), mean (SD)	69.5 (15.9)	67.5(10.5)	72.5 (12.8)	0.357
Gender				0.065
Female, *n* (%)	41 (62.1)	8 (38.1)	19 (70.4)	
Male, *n* (%)	25 (37.9)	13 (61.9)	8 (29.6)	
Comorbidity score, mean (SD)	14.7 (10.4)	10 (8.4)	14.4 (13.1)	0.213
*Type of infection*				
Hospital acquired, *n* (%)	21 (31.8)	1 (4.8)	8 (29.6)	0.046
Healthcare associated^∗^, *n* (%)	21 (31.8)	6 (28.6)	10 (37)	0.814
Community acquired, *n* (%)	24 (36.4)	13 (61.9)	9 (33.3)	0.082
*Source of infection*				
Catheter LSI, *n* (%)	0 (0)	1 (4.8)	0 (0)	0.109
Bacteremia, *n* (%)	4 (6.1)	2 (9.5)	3 (11.1)	0.684
Septic shock, *n* (%)	8 (12.1)	2 (9.5)	5 (18.5)	0.614
Intra-abdominal, *n* (%)	5 (7.6)	0 (0)	2 (7.4)	0.434
Pneumonia, *n* (%)	7 (10.6)	1 (4.8)	1 (3.7)	0.452
SSTI, *n* (%)	10 (15.2)	6 (28.6)	2 (7.4)	0.136
UTI, *n* (%)	44 (66.7)	12 (57.1)	19 (70.4)	0.618
*Culture species*				
*E. coli*, *n* (%)	51 (77.3)	18 (85.7)	24 (88.9)	0.369
*K. pneumoniae*, *n* (%)	11 (16.7)	1 (4.8)	1 (3.7)	0.118
*P. mirabilis*, *n* (%)	15 (22.7)	5 (23.8)	0 (0)	0.024

^∗^Healthcare-associated infections = acquired in nursing resident or recent hospitalization.

**Table 2:** Primary and secondary outcomes.

Outcome	Carbapenem (*n* = 66)	Cefepime (*n* = 21)	Levofloxacin (*n* = 26)	*p* value
*Primary*				
Expired patients in hospital, *n* (%)	11 (16.6)	0 (0)	4 (15.4)	0.253
*Secondary*				
Expired patients in ICU, *n* (%)	3 (4.5)	0 (0)	1 (3.7)	0.616
ICU length of stay, mean (SD)	9.8 (16.0)	7.8 (6)	5.4 (4.1)	0.805
Hospital length of stay, mean (SD)	12.1 (11)	11.1 (10.5)	11.1 (10.4)	0.685

**Table 3:** Multivariate analysis.

	Mortality		Unadjusted OR	Adjusted OR	*p*
	No (*n* = 99)	Yes (*n* = 15)			
Vascular catheter LSI	1 (1.0)	0 (0.0)	—	—	—
CLSI	0 (0.0)	0 (0.0)	—	—	—
Ventilator-related infection	0 (0.0)	0 (0.0)	—	—	—
Bacteremia	8 (8.1)	1 (6.7)	0.81 (0.02–6.92)	0.63 (0.07–6.08)	0.692
Sepsis shock	14 (14.1)	1 (6.7)	0.43 (0.01–3.34)	0.31 (0.03–3.00)	0.314
Intra-abdominal infection	7 (7.1)	0 (0.0)	0.00 (0.00–3.57)	—	—
Pneumonia	6 (6.1)	3 (20.0)	3.87 (0.54–20.9)	2.28 (0.26–20.15)	0.458
Skin and soft tissue infection	17 (17.2)	1 (6.7)	0.34 (0.01–2.59)	0.26 (0.02–2.97)	0.278
Urinary tract infection	66 (66.7)	9 (60.0)	0.75 (0.21–2.79)	0.53 (0.10–2.84)	0.461
